# Supplementary material for: Bone Marrow-Suppressive Treatment in Children Is Associated with Diminished IFN-γ Response from T Cells upon Polyclonal and Varicella Zoster Virus Peptide Stimulation
Source: Int J Mol Sci. 2024 Jun 26;25(13):6960. doi: 10.3390/ijms25136960 (PMC11241059; doi:10.3390/ijms25136960)
Supplement: Supplementary file 1 [file ijms-25-06960-s001.zip › Figure legend.pdf]

**Figure legends:**

**Supplementary figure 1.** Variations in regulatory T-cell populations in peripheral venous blood among the three groups. Live regulatory T cells were characterised as $DCM^{-}CD4^{+}CD25^{hi}CD127^{-/low}FOXP3^{+}$ lymphocytes. A Kruskal-Wallis, with Dunn's multiple comparison test, was used to determine significant differences. Statistical significance was determined as $p<0.05$ and the median is shown as a red horizontal line.

**Supplementary figure 2.** Frequency of IFN-γ producing cells in response to T-cell mitogen, Staphylococcal enterotoxin A (SEA) and Varicella Zoster Virus (VZV) peptides IE63 and gE, in children with bone-marrow suppressive treatment (group III), according to allogeneic-haematopoietic stem cell transplantation (allo-HSCT). (A) Differences in number of IFN-γ spot-forming cells (SFC) between children with bone-marrow suppressive treatment having undergone allo-HSCT or not (SEA n=17, VZV n=15). (B) Differences in IFN-γ residual spot volume (RSV) between children in group III having undergone allo-HSCT or not (SEA n=17, VZV n=15). Horizontal red lines represent median, points in pink represent children having received chimeric antigen receptor (CAR)-T cells, points in blue children with severe haematological disease, remaining points in black represent children with lymphoid malignancies.
