# Supplementary figures and images for: Bone Marrow-Suppressive Treatment in Children Is Associated with Diminished IFN-γ Response from T Cells upon Polyclonal and Varicella Zoster Virus Peptide Stimulation
Source: Int J Mol Sci. 2024 Jun 26;25(13):6960. doi: 10.3390/ijms25136960 (PMC11241059; doi:10.3390/ijms25136960)

**Live CD4+ T cells**

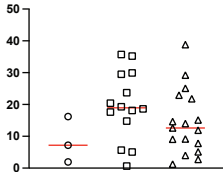

**CD4+CD25+CD127-**

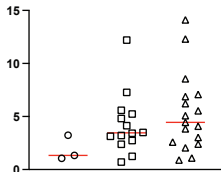

**CD4+CD25+CD127-FOXP3+**

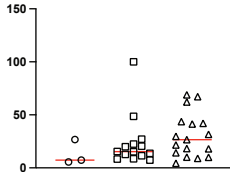

○ **Group I**  
□ **Group II**  
△ **Group III**

Supplement: Supplementary file 1 [file ijms-25-06960-s001.zip › Suppl fig S1.pdf]

**A**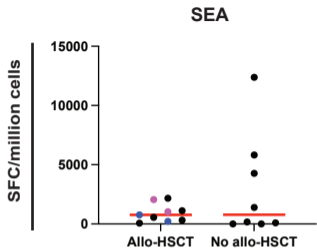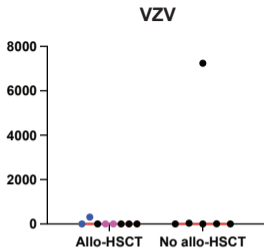**B**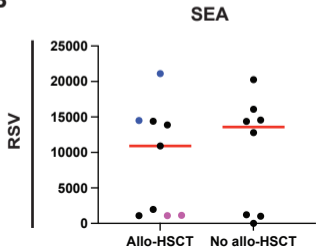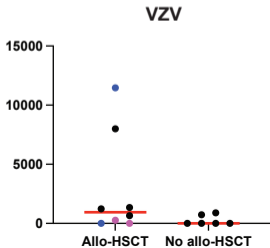

Supplement: Supplementary file 1 [file ijms-25-06960-s001.zip › Suppl fig S2.pdf]
